# Supplementary material for: Nanosensor Based on Thermal Gradient and Machine Learning for the Detection of Methanol Adulteration in Alcoholic Beverages and Methanol Poisoning
Source: Sensors (Basel). 2022 Jul 25;22(15):5554. doi: 10.3390/s22155554 (PMC9329758; doi:10.3390/s22155554)
Supplement: Supplementary file 1 [file sensors-22-05554-s001.zip › sensors-1819778-supplementary.pdf]

Supplementary Material

Euclidean UPGMA clustering

Euclidean clustering was used to confirm the good classification obtained with the LDA in all humidity conditions.

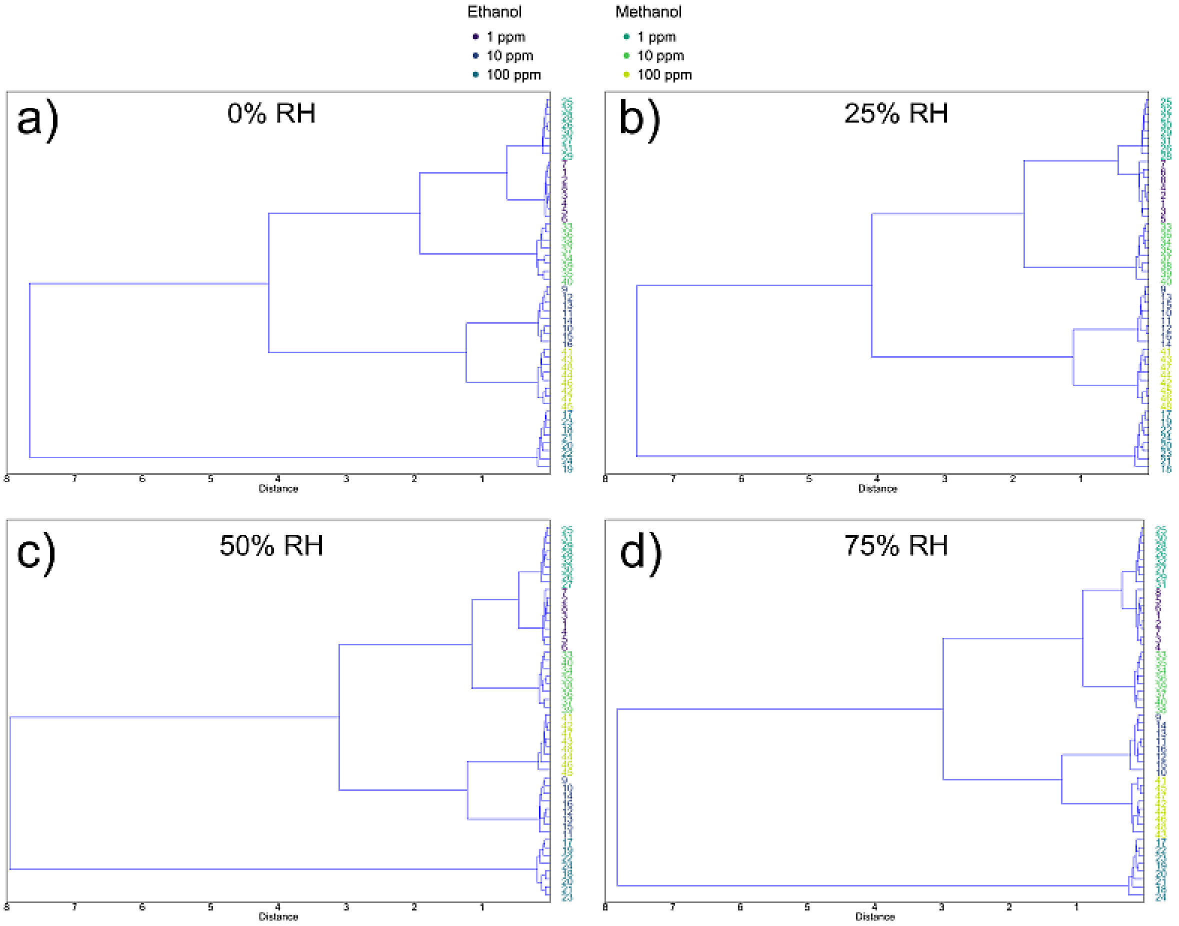

Figure S1. UPGMA dendrograms obtained in different humidity conditions: a) 0%, b) 25%, c) 50% and d) 75%.

The four dendrograms in Fig. S1 confirm the goodness of the classification obtained with LDA. As you can see on the right, the samples are grouped according to the type of gas and its concentration.
